# Endoscopic, laboratory, and clinical findings and outcomes of caustic ingestion in adults; a retrospective study 

**Published:** 2022

**Authors:** Ali Banagozar Mohammadi, Maryam Zaare Nahandi, Ali Ostadi, Anahita Ghorbani, Shahin Hallaj

**Affiliations:** 1 *Medical Philosophy and History Research Center, Tabriz University of Medical Sciences, Tabriz, Iran*; 2 *Kidney Research Center, Tabriz University of Medical Sciences, Tabriz, Iran*; 3 *Department of Internal Medicine, School of Medicine, Tabriz University of Medical Sciences, Tabriz, Iran*; 4 *Student Research Committee, Tabriz University of Medical Sciences, Tabriz, Iran*; 5 *Immunology Research Center, Tabriz University of Medical Sciences, Tabriz, Iran*

**Keywords:** caustic, clinical, endoscopy, ingestion, outcome

## Abstract

**Aim:**

Compared to the prevalence and complications, there is still limited evidence in this regard.

**Background:**

With an incidence rate of 200,000 cases annually and the induction of numerous complications, caustic ingestion imposes a significant burden on the healthcare system. Apart from being fatal in some cases, this injury affects its victims’ quality of life as it is followed by many gastrointestinal problems. This injury mainly occurs accidentally among children, whereas in adults, it often occurs with suicidal intentions. Despite recent advances in internal medicine, gastroenterology, and toxicology, this type of injury remains a debilitating and, in some cases, fatal disorder for its victims.

**Methods:**

This study retrospectively evaluated the clinical, laboratory, and endoscopic findings of 150 patients admitted to a referral center of toxicology and forensic medicine and assessed factors associated with each type of injury.

**Results:**

The findings indicated a mortality rate as high as 7.3% in this population. Age, pH, and previous medical conditions were associated with more complications. Higher degrees of injury were also significantly associated with higher mortality. No significant difference was observed between types of corrosive substances.

**Conclusion:**

It seems that the most effective intervention for controlling caustic ingestion injuries would be psychiatric support, primary healthcare, and household education.

## Introduction

 Corrosive or caustic substances are chemicals that harm tissue in case of exposure. Although the most frequently affected organs are the face, eyes, and extremities, mortal cases are highly frequent following caustic ingestion (1, 2). According to the American Association of Poison Controls, more than 200,000 corrosive ingestions occur annually, commonly caused by the abuse of acids, alkalis, formalin, heavy metal salts, and chemical agents. Most caustic intoxication cases are suicidal, and most cases involve women (3). 

The substances mentioned above could induce some injury, especially in narrowing areas of the esophagus (i.e., middle and distal parts), antrum, and pylorus, leading to different complications (e.g., stricture, esophageal carcinoma, mediastinitis) and even death (4). According to the literature, the most prevalent complications following caustic ingestion are esophageal and gastric stenosis, usually occurring after the ingestion of acid. Clinical manifestations include pain in the mouth, retrosternal pain, hypersalivation, odynophagia, dysphagia, and hematemesis (5). 

Caustic ingestion usually induces one of two types of injuries, acute or chronic. Acute phase injury includes inflammation, necrosis, and gangrene, and in this situation, medical interventions mainly control the perforation, secure the airway, provide fluid resuscitation, and manage the possible shock (6). However, the chronic phase involves bleeding, fibrosis, and strictures, and interventions aim to correct the stenosis, repair the formed fistulas, and manage the ingestion disorders following esophageal movement disorders and management of the esophageal diverticulitis (7). In cases of gastrointestinal perforation (e.g., mediastinitis, peritonitis), urgent surgery is indicated; otherwise, management is primarily based on the findings of an esophago-gastro-duodenoscopy procedure and, in some cases, endoscopic ultrasound (EUS) or abdominal computed tomography (CT) scan to evaluate the depth of the injury (8).

Despite recent advances in primary healthcare, toxicology, and gastroenterology, caustic injuries and the morbidity and mortality caused by them are still a significant issue in toxicology, surgery, and internal medicine wards. This study evaluated the demographic features and clinical manifestations of patients who ingested corrosive substances as well as the substances’ contribution to the mortality of patients admitted to a referral center for toxicology, internal medicine, surgery, and clinical forensic medicine in one year. 

## Methods

The medical records of patients admitted to a referral center for internal medicine, surgery, medical toxicology, and clinical forensic medicine, who underwent endoscopy during their admission following caustic ingestion in one year were evaluated. After applying the inclusion and exclusion criteria, 150 patients were included in this study. The evaluated parameters included age, sex, caustic substance type and amount ingested, cause of ingestion, ingestion-admission interval, and laboratory tests. 

The endoscopic studies were performed by a single board-certified professor of gastroenterology with more than ten years of clinical, educational, and research experience, and the clinical records of other operators were excluded from the study. 

**Inclusion criteria**

- Ingestion of caustic substances
- Undergoing endoscopy during admission
- Complete and consistent medical records
- Patient’s consent

**Exclusion criteria**

- Incomplete medical records
- Caustic injury without ingestion of the caustic substance
- Patient’s refusal to participate in this study

**Informed consent**

Informed consent to use their clinical, laboratory, and unidentified personal data for publication and scholar purposes was obtained from all individual participants included in the study. 

***Statistical analysis***

Data was analyzed by descriptive statistics, t-test, and chi-squared test using (SPSS 26; IBM SPSS Statistics) and reported in tables. Statistical significance was set at *p*≤0.05. 

## Results

**Demographic findings**

**Figure 1:**
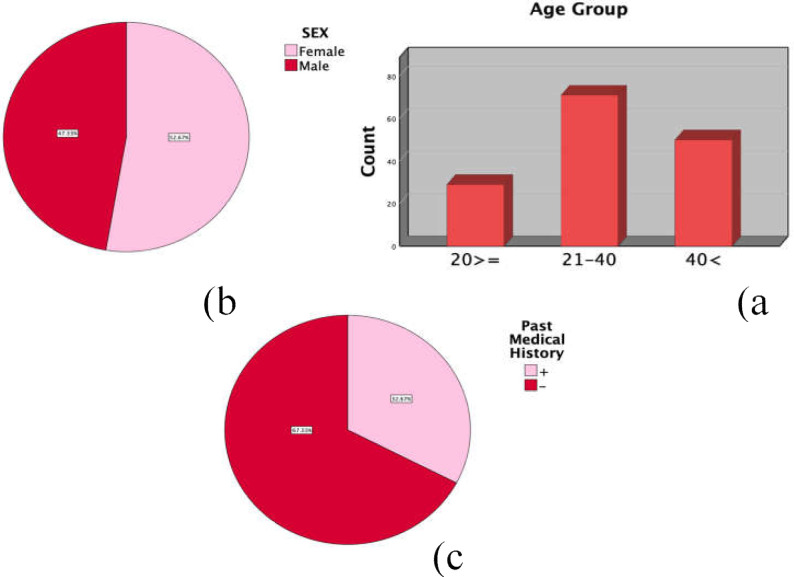
a: Bar chart demonstrating the frequency patients in each group of age, b: Pie chart indicating groups of sex, c: Pie chart demonstrating the prevalence of previous medical conditions among cases

Of 150 patients, 52.7% were female and 47.3% were male; 19.3% of patients were younger than 20 years of age, 47.3% and 33.3% were 21‒40 and >41 years of age, respectively, and 32.7% of all patients had a history of medical conditions (Figure 1).

**Features of the caustic injury**

As shown in Table 1, 40.7% of patients had no pathological findings in their endoscopic study. However, 36%, 19.3%, and 4% of the patients had first-, second-, and third-degree burns in their gastrointestinal tract. Of all injuries, 32% were caused by sodium hypochlorite (NaOCl) solution, followed by depilatory agents, hydrochloric acid, sulfuric acid, drain cleaner, sodium hydroxide, paraquat, and other corrosive substances (e.g., hydrogen peroxide, carpet cleaning shampoo, dishwasher tablets, and washing powders) as the less prevalent causes of injury. Based on the notes documented in the patients’ medical files and regarding the pH of the substances, 44.7% were acidic, 43.3% were alkaline, and the pH of 12% of the substances was unknown and unrecorded. Seventy-two percent of the ingestions were intended to commit suicide, and 70.7% were performed between 12 pm and 12 am. Moreover, 48.5% of cases were admitted to the center <2 hours after ingestion, 40.7% were admitted between 2 to 6 hours, and 11.3% were admitted into our center >6 hours after the caustic ingestion incident. Additionally, 41.3% of patients had one or two complaints, whereas 58.7% complained of more than two clinical conditions. Among all participants, 68.7% had abnormal laboratory tests, and 7.3% of all patients died during their hospital stay. The most prevalent manifestations of the studied cases were parallel skin injuries and abdominal pain (Table 1), and the most prevalent objective finding was epigastric tenderness. 

**Table 1:** Demographic features of the population

Parameter		Count (n=150)	%
Endoscopic findings/grade of injury	Normal	61	40.7
	1st degree	54	36.0
	2nd degree	29	19.3
	3rd degree	6	4.0
Caustic substance	Depilatory agents	43	28.7
	Sulfuric acid	11	7.3
	Sodium hydroxide	7	4.7
	Sodium hypochlorite	48	32.0
	Hydrochloric acid	12	8.0
	Drain cleaner	10	6.7
	Paraquat	3	2.0
	Other corrosive substances	16	10.7
Intention	Intentional	108	72.0
	Unintentional	42	28.0
Injury-admission interval	< 2 h	72	48.0
	2-6 h	61	40.7
	> 6 h	17	11.3
Number of symptoms	1 or 2 symptoms	62	41.3
	>2 symptoms	88	58.7
Lab tests	Normal	47	31.3
	Abnormal	103	68.7
Lab	Normal	47	31.3
	Anemia	58	38.7
	Acid-Base disturbance	11	7.3
	Renal injury	15	10.0
	Liver injury	0	0
	Coagulopathy	6	4.0
	Hypoglycemia	0	0
	Leukocytosis	58	38.7
Outcome	Lived	139	92.7
	Dead	11	7.3
Esophageal endoscopy	Normal	97	64.7
	Abnormal	53	35.3
Gastric endoscopy	Normal	94	62.7
	Abnormal	56	37.3
Duodenal endoscopy	Normal	118	78.7
	Abnormal	32	21.3
Signs and Symptoms	Sialorrhea	10	6.7
	Vomiting	71	47.3
	Abdominal pain	85	56.7
	GI-Bleeding	27	18.0
	Dysphagia	23	15.3
	Odynophagia	52	34.7
	Burning wounds (Face, mouth, & throat)	87	58.0
	Tenderness in epigastric area	65	43.3
	Dyspnea	27	18.0
	Pulmonary rales	8	5.3
	Normal	6	4.0

**Table 2:** Factors associated with esophageal endoscopic findings

Parameters		Esophageal endoscopic findings				*P*-value
		Normal		Abnormal		
Sex	Female	52	65.8%	27	34.2%	.755
	Male	45	63.4%	26	36.6%	
Age	<20	27	93.1%	2	6.9%	.002*
	21-40	41	57.7%	30	42.3%	
	>40	29	58.0%	21	42.0%	
Past medical history	Yes	35	71.4%	14	28.6%	.228
	No	62	61.4%	39	38.6%	
Intention	Intentional	71	65.7%	37	34.3%	.659
	Unintentional	26	61.9%	16	38.1%	
Injury-admission interval	<2 h	45	62.5%	27	37.5%	.852
	2-6 h	41	67.2%	20	32.8%	
	>6 h	11	64.7%	6	35.3%	
Number of symptoms	1-2 symptoms	44	71.0%	18	29.0%	.175
	>2 symptoms	53	60.2%	35	39.8%	
Lab Tests	Normal	33	70.2%	14	29.8%	.337
	Abnormal	64	62.1%	39	37.9%	
Outcome	Lived	92	66.2%	47	33.8%	.166
	Dead	5	45.5%	6	54.5%	

**Table 3:** Factors associated with gastric endoscopic findings

Parameters		Gastric endoscopic findings				*P*-value
		Normal		Abnormal		
Sex	Female	45	57.0%	34	43.0%	.128
	Male	49	69.0%	22	31.0%	
Age	<20	23	79.3%	6	20.7%	.015*
	21-40	47	66.2%	24	33.8%	
	>40	24	48.0%	26	52.0%	
Past medical history	Yes	25	51.0%	24	49.0%	.040*
	No	69	68.3%	32	31.7%	
Intention	Intentional	63	58.3%	45	41.7%	.078
	Unintentional	31	73.8%	11	26.2%	
Injury-admission interval	<2 h	44	61.1%	28	38.9%	.522
	2-6 h	41	67.2%	20	32.8%	
	>6 h	9	52.9%	8	47.1%	
Number of symptoms	1-2 symptoms	36	58.1%	26	41.9%	.328
	>2 symptoms	58	65.9%	30	34.1%	
Lab Tests	Normal	29	61.7%	18	38.3%	.869
	Abnormal	65	63.1%	38	36.9%	
Outcome	Lived	85	61.2%	54	38.8%	.173
	Dead	9	81.8%	2	18.2%	

**Table 4:** Factors associated with duodenal endoscopic findings

Parameters		Duodenal endoscopic findings				*P*-value
		Normal		Abnormal		
Sex	Female	64	81.0%	15	19.0%	0.459
	Male	54	76.1%	17	23.9%	
Age	<20	28	96.6%	1	3.4%	.020*
	21-40	55	77.5%	16	22.5%	
	>40	35	70.0%	15	30.0%	
Past medical history	Yes	42	85.7%	7	14.3%	0.142
	No	76	75.2%	25	24.8%	
Intention	Intentional	87	80.6%	21	19.4%	0.365
	Unintentional	31	73.8%	11	26.2%	
Injury-admission interval	<2 h	59	81.9%	13	18.1%	0.230
	2-6 h	44	72.1%	17	27.9%	
	>6 h	15	88.2%	2	11.8%	
Number of symptoms	1-2 symptoms	51	82.3%	11	17.7%	0.367
	>2 symptoms	67	76.1%	21	23.9%	
Lab Tests	Normal	35	74.5%	12	25.5%	0.396
	Abnormal	83	80.6%	20	19.4%	
Outcome	Lived	108	77.7%	31	22.3%	0.303
	Dead	10	90.9%	1	9.1%	

Nonetheless, the highest mortality rate was observed in the bleach group (12.5% of cases) and acidic substances overall (10.4% of cases).

**Laboratory and endoscopic findings**

Of all patients, 31.3% had normal laboratory tests, and the most prevalent laboratory findings were anemia (38.7%), leukocytosis (38.7%), renal injury (10%), acid-base disorders (7.3%), and coagulopathy (4%) in descending order. 

Furthermore, 35.3%, 37.3%, and 21.3% of patients had esophageal, gastric, and duodenal injuries as revealed by endoscopy. As shown in Tables 2, 3, and 4, advancing age significantly correlated with the esophageal, gastric, and duodenal injury (*p*<0.05). Additionally, previous medical conditions made the patients more susceptible to gastric injuries (*p*<0.05).

**Assessment of measures taken before admission**

Among the 150 assessed patients, 32 (21.3%) had undergone incorrect interventions, including vomiting induction (n=26, 17.33%), charcoal prescription (n=3, 2%), and unknown neutralizing substance use (n=3, 2%) prior to admission. Fortunately, no incorrect measures had been taken in the remaining 118 (79%) cases.

**Expired patients**

Of all the 150 patients assessed herein, 11 cases (i.e., five women and six men) died. As shown in Table 5, no significant associations were found between mortality, sex, age, previous medical history (PMH), site of injury, ingestion-admission period, laboratory tests, and type of abused corrosive substance. However, higher grades of injury were significantly associated with a greater risk of mortality among cases. All of the expired patients had a 2nd or 3rd degree injury, and the mortality rate of patients with normal or first-degree injury endoscopic findings was zero. Moreover, among reported presenting signs and symptoms, dysphagia and pulmonary rales were significantly associated with increased risk of mortality among patients.

**Table 5:** Effect of the measured parameters on case mortality following caustic ingestion. *P*-values and odds ratios are calculated using binary logistic regression analysis

Parameter		Outcome					
		Lived		Expired			
		Count	Row N%	Count	Row N%	*P*-value	Odds ratio
Grade of injury	Normal	61	100.0%	0	0.0%	0.021*	1718.507
	I	54	100.0%	0	0.0%		
	II	23	79.3%	6	20.7%		
	III	1	16.7%	5	83.3%		
PMH	Yes	46	93.9%	3	6.1%	0.266	16.105
	No	93	92.1%	8	7.9%		
Sex	Female	74	93.7%	5	6.3%	0.370	0.172
	Male	65	91.5%	6	8.5%		
Age (y)	20=>	25	86.2%	4	13.8%	0.641	1.574
	20<x<=40	68	95.8%	3	4.2%		
	40<	46	92.0%	4	8.0%		
Esophagus	Normal	92	94.8%	5	5.2%	0.083	40.044
	Injured	47	88.7%	6	11.3%		
Stomach	Normal	85	90.4%	9	9.6%	0.599	2.708
	Injured	54	96.4%	2	3.6%		
Duodenum	Normal	108	91.5%	10	8.5%	0.513	0.007
	Injured	31	96.9%	1	3.1%		
Ingestion-admission period (h)	<2	67	93.1%	5	6.9%	0.137	6.017
	2<=x<6	57	93.4%	4	6.6%		
	6<x	15	88.2%	2	11.8%		
Lab tests	Normal	45	95.7%	2	4.3%	0.162	31.793
	Disturbed	94	91.3%	9	8.7%		
Type of corrosive substance	Acidic	60	89.6%	7	10.4%	0.852	1.074
	Alkaline	62	95.4%	3	4.6%		
	Unknown	17	94.4%	1	5.6%		

## Discussion

Ingestion of caustic agents usually occurs accidentally among children (9, 10); however, in case of adults, as in the present study, ingestion is usually associated with suicidal intentions. This type of suicide is more prevalent among women than men and causes acute and chronic complications. Mortality following caustic ingestion is as high as 5‒10% (11), consistent with the present study, in which mortality was calculated at 7.3%. 

According to the literature, the most abused substance in cases of caustic injuries is toilet cleaner, which contains strong acid of alkali (12, 13). However, in the current study, the most commonly abused substance was bleach (sodium hypochlorite). Accordingly, the highest mortality rate was observed in the bleach group.

There are still many controversies regarding the association between presenting signs and symptoms and poor prognosis (12). Drooling of saliva and sialorrhea have been reported as the predictors of higher grades of mucosal injury (13). In the present study, dysphagia and pulmonary rales were significantly associated with a greater risk of mortality, though no significant association was found between presenting signs and symptoms and the grade of injury.

In the present study, anemia was the most prevalent laboratory finding among the patients, which could be considered endemic prevalent anemia and/or gastrointestinal bleeding. Second to anemia, leukocytosis is the most reported laboratory finding among cases, consistent with the present study, and might be attributed to inflammation and stress. Additionally, as in the literature (1, 4), esophageal injuries were the most prevalent, followed by gastric and duodenal wounds. 

Age, pH, and previous medical conditions were associated with more complications, and it seems that the most effective intervention for controlling these types of injuries would be psychiatric support, primary healthcare, and household education. As mentioned above, a significant number of cases had undergone improper interventions before coming to the center and being admitted. To address this error, it is recommended that continuous educative materials be provided for both ordinary members of society and members of medical care teams. 

Moreover, a higher degree of injury was significantly associated with higher mortality. However, in the literature, injuries of a higher degree were significantly associated with the abused substances’ pH. In the present study, no significant difference was observed between types of corrosive substances; however, the pH of substances was not evaluated.

All the expired patients had a 2nd or 3rd degree injury, and the mortality rate of patients with normal or first-degree injury endoscopy findings was zero. Overall, the current findings are consistent with those of previous studies. Nonetheless, more standardized studies with a larger sample size and control cases are strongly recommended.

## Conflict of interests

The authors declare that they have no conflict of interest.
